# Combination of albumin-lymphocyte score and skeletal muscle index predicts prognosis of patients with ovarian cancer after primary debulking surgery

**DOI:** 10.3389/fimmu.2026.1781291

**Published:** 2026-04-15

**Authors:** Yihong Chen, Quanmin Kang, Dan Nie, Peng Zhang, Xiaoxia Zhou, Zhaoci Li, Yulan Hu, Tan Xin, Han Gong

**Affiliations:** 1 Key Laboratory of Birth Defects and Related Diseases of Women and Children, Ministry of Education, West China Second University Hospital, Sichuan University, Chengdu, Sichuan, China; 2 Department of Obstetrics and Gynecology, West China Second University Hospital, Sichuan University, Chengdu, Sichuan, China; 3 Department of Day Surgery, West China Second University Hospital, Sichuan University, Chengdu, Sichuan, China; 4 Key Laboratory of Reproductive Genetics of National Ministry of Education, Women’s Reproductive Health Laboratory of Zhejiang Province, Women’s Hospital, School of Medicine, Zhejiang University, Hangzhou, Zhejiang, China; 5 Department of Reproductive Endocrinology, Women’s Reproductive Health Laboratory of Zhejiang Province, Women’s Hospital, School of Medicine, Zhejiang University, Hangzhou, Zhejiang, China; 6 Department of Gynecology, The Affiliated Traditional Chinese Medicine Hospital, Southwest Medical University, Luzhou, Sichuan, China; 7 Department of Gynecology and Obstetrics, People’s Hospital of Leshan, Leshan, Sichuan, China; 8 Department of Gynecology, Panzhihua Central Hospital, Panzhihua, Sichuan, China; 9 Department of Gynecology, Affiliated Hospital of Zunyi Medical University, Zunyi, Guizhou, China; 10 Department of Obstetrics and Gynecology, Wan’an Maternal & Child Health Care Hospital, Wan’an, Jiangxi, China

**Keywords:** albumin-lymphocyte score, debulking surgery, ovarian cancer, prognosis, skeletal muscle index

## Abstract

**Purpose:**

Accumulating evidence underscores the significance of inflammation and nutrition in tumor progression. Although low albumin-lymphocyte score (ALS) and skeletal muscle index (SMI) are known to be associated with negative outcomes in patients with ovarian cancer (OC) undergoing primary debulking surgery, the usefulness for predicting prognosis remains unclear. We aimed to assess the relevant preoperative prognostic variables and their combined impact on patients with OC.

**Methods:**

This retrospective study included 347 patients with primary OC from multiple medical centers. The patients were divided into discovery (237 patients) and validation (110 patients) cohorts. Serological tests and plain computed tomography were performed to quantify the ALS and SMI. We investigated the preoperative prognostic ability of a unique index based on a combination of ALS and SMI, the CAS grade.

**Results:**

Patients with a lower ALS and a higher SMI showed improved overall survival (OS) and recurrence-free survival (RFS). Upon stratification by CAS grade, grade 1 patients demonstrated the highest body mass index and the most favorable survival prognosis, whereas grade 3 patients demonstrated the poorest OS and RFS. The independent variables for OS and RFS included residual disease and elevated CAS grade. These findings were validated in an independent cohort study.

**Conclusion:**

The CAS grade, a combination of ALS and SMI, is a meaningful and independent predictor of prognosis in patients with OC.

## 1 Introduction

Among all gynecological cancers, ovarian cancer (OC) ranks second globally in terms of mortality rates, and it is the eighth most frequent cancer in women, giving rise to approximately 295,414 newly diagnosed patients and 184,799 deaths in 2018 (1). OC is generally divided into epithelial OC (EOC) and non-epithelial OC, with EOC accounting for nearly 90% of all primary OC cases and demonstrating a rising mortality rate in recent years. Unlike non-epithelial tumors, epithelial tumors are more malignant with insidious early symptoms, and most patients are diagnosed at an advanced stage with the spread of disease outside the pelvis (2). Among all therapeutic management strategies for EOC, including debulking surgery, neoadjuvant chemotherapy, postoperative platinum-based chemotherapy, and targeted therapies, surgical treatment is still considered the gold standard for newly diagnosed EOC (3).

Extensive studies have focused on the prediction of the prognosis of patients with OC (4, 5). The revised FIGO staging system combines the classification for ovarian, fallopian tube, and peritoneal cancers and updates the classification of stages based on surgical exploration. In the current 2014 FIGO staging classification, the IIIA stage is further subdivided into IIIA1 and IIIA2 according to the greatest dimension of metastatic focus (6). In addition to the traditional tumor staging system, serum biomarkers, imaging manifestations, and their combined statistical models have been explored to predict the prognosis of diagnosed EOC.

Malnutrition, a clinical condition commonly seen in multiple cancers, is associated with poor survival prognosis, reduced treatment completion, and high healthcare consumption (7). In general, nutrition is considered to indirectly affect tumorigenesis, which has a profound influence on leukocytes, thereby altering pro-inflammatory carcinogenic effects or anticancer immune responses (8). Previous studies have proven that nutrition-based serum biomarkers, such as the albumin-globulin ratio, albumin-globulin score, prognostic nutritional index (PNI), and Glasgow Prognostic Score (GPS), are independent factors for predicting the prognosis of multiple cancers (9, 10). Recently, a combination of lymphocyte count and albumin concentration was found to significantly correlate with poor overall survival (OS) and recurrence-free survival (RFS) in rectal cancer (11). However, the predictive value of the albumin-lymphocyte score (ALS) and survival of patients with OC after debulking surgery awaits further investigation.

In clinical practice, malnutrition is considered a muscle-related disorder (12). Proper assessment of the skeletal muscle is also a fundamental and mandatory part of the evaluation of prognosis in patients with cancer. Plain computed tomography (CT) is a noninvasive technology that is commonly used in the clinical setting to measure muscle mass (13). The skeletal muscle index (SMI) has been used in earlier studies to predict survival in patients with OC (14).

Therefore, the aim of this study was to determine how ALS and SMI affect the survival of patients with OC after primary surgery. Additionally, a unique index based on a combination of the SMI and ALS (CAS) was developed to evaluate the nutritional status of patients with OC following debulking surgery more thoroughly in terms of both serological and imaging features. Following debulking surgery, the impact of CAS grade on OS and RFS in patients with OC was also examined.

## 2 Patients and methods

### 2.1 Patient baseline characteristics

In this retrospective investigation conducted across multiple medical centers, 237 patients with surgically-managed EOC treated at West China Hospital between September 2015 and December 2018, were enrolled. The patient cohort from West China Hospital served as the basis for investigating our initial statistical model (discovery cohort). Validation of our observations was performed in a separate cohort (comprising 110 patients) drawn from additional healthcare institutions, including Leshan Hospital, The First Affiliated Hospital of Zunyi Medical University, and Panzhihua Central Hospital. This study was approved by the Institutional Ethics Committee of the West China Second Hospital (2022 Medical Scientific Research for Ethical Approval No.167). Consent to participate was not necessary as the data were fully anonymized, according to our privacy and ethical policy. All patient information obtained from hospitals was anonymous, and all primary cytoreduction procedures were undertaken in all enrolled patients, all of whom received a histopathological diagnosis of EOC. The following individuals were excluded from our study: (1) those with autoimmune, hematological, or infectious diagnoses; (2) those who did not undergo a preoperative blood routine examination; and (3) those who did not undergo preoperative plain CT.

### 2.2 Clinical information aggregation and endpoints

The comprehensive examinations, retrieval of patient data, and preoperative serological assessments were conducted by reviewing electronic and handwritten medical records at the hospital. Weight and height measurements were gathered to compute body mass index (BMI) based on the following formula: weight (kg)/height (m^2^). For the statistical analysis of the training cohort, the ideal cut-off values for albumin (Alb) and lymphocyte count were found to be 43.90 g/L and 1.34×10^9^/L, respectively, using the X-tile 3.6.1 program (Yale University, New Haven, CT, USA) (15). An Alb value ≥43.90 g/L and a lymphocyte count ≥1.34×10^9^/L were considered an increase. According to the findings in earlier research, the ALS was delineated as follows: patients with normal values for both Alb (≥43.90 g/L) and lymphocyte count (≥1.34×10^9^/L) were assigned as ALS0, and those with hypoalbuminemia (<43.90 g/L) and a reduced lymphocyte count (<1.34×10^9^/L) were classified as ALS2, whereas individuals with only one of the two abnormalities were categorized as ALS1. Additionally, we collected data on clinicopathological features pertinent to the tumors, including age, histology, grade, laterality, residual disease, peritoneal cytology, postoperative chemotherapy, lymphadenectomy, lymph node metastasis (LNM), and pre-operative CA125. Staging adhered to the guidelines established by the International Federation of Gynecology and Obstetrics in 2018 (6). OS represents the period from curative resection to either death or last follow-up for patients without recurrence. RFS represents the period from curative resection to recurrence identification or the last follow-up for patients without recurrence.

### 2.3 Imaging analysis

Before initial surgery, all patients underwent plain CT. The calculation of the skeletal mass region was completed on transverse, non-contrast plain CT scans at the level of the third lumbar vertebra using Mimics software (version 21.0; Materialise NV, Leuven, Belgium). The transverse region of the skeletal muscle (cm²) divided by height (m²) was computed as the SMI (13). Through statistical analysis of the training cohort, X-tile 3.6.1 software (Yale University) identified the best cut-off value for SMI (15). The high SMI group was deemed as those with an SMI ≥38.53 cm²/m. Subsequently, the CAS grade was characterized as follows: CAS degree 1 was assigned to individuals presenting with both low ALS (0) and high SMI, CAS degree 3 was assigned to individuals presenting with both high ALS (1/2) and low SMI, and CAS degree 2 was assigned to all others (13).

### 2.4 Statistical analysis

Statistical analyses were performed using Stata 14.0 software (StataCorp LP; College Station, TX, USA), MedCalc (version 15.2.2.0; Ostend, Belgium), and GraphPad Prism (version 9.0; San Diego, CA, USA). Receiver operating characteristic (ROC) curves were used to compare discrepancies among the areas under the curve (AUC), and a 4-year survival time was established as the endpoint. For each cut-off value, Kaplan–Meier curves were constructed for the validation and discovery cohorts based on each cut-off value, and the log-rank test was employed to compare the discrepancies between cohorts. The AUCs for the ALS, SMI, and CAS were also calculated and compared. To identify the putative independent prognostic factors for OS and RFS, a Cox proportional hazards regression model was used to perform univariate and multivariate analyses. Statistical significance was set at a two-tailed P value of <0.05.

## 3 Results

### 3.1 Discovery cohort

The basic statistics of included individuals in training cohort (n=237) are described in **Table 1**. The median age of participants was 50.2 (15–79 years). The median BMI and SMI were 22.9 kg/m^2^ and 41.75 cm^2^/m^2^, respectively. Based on the comprehensive assessment of the patients’ general condition, 31 (13.1%) patients did not undergo systemic pelvic and para-aortic lymphadenectomy; thus, we could not validate their precise FIGO stage. Eighty-three (35.1%) and 154 (64.9%) patients were categorized into the low-ALS and high-ALS group, respectively.

**Table 1:** The baseline characteristics of patients in the training and validation cohorts.

Patient characteristics	Training cohort		Validation cohort	
	N=237	%	N=110	%
Age [year, mean(range)]	50.2(15-79)		52.5(18-78)	
BMI [mean(range)]	22.9(13.6-32.6)		23.1(15.9-32.0)	
SMI [mean(range)]	41.8(27.8-67.5)		38.6(24.6-53.7)	
Histology				
Serous	137	57.8	85	77.3
Others	100	42.2	25	22.7
Grade				
1-2	51	21.5	31	28.2
3.0	186	78.5	79	71.8
FIGO stage				
IA-IIB	103	43.5	53	48.2
III-IV	103	43.5	57	51.8
Unknown	31	13.0	0	0.0
Laterality				
Unilateral	159	67.1	78	70.9
Bilateral	78	32.9	32	29.1
Residual disease				
<1cm	209	88.2	86	78.2
≥1cm	28	11.8	24	21.8
Peritoneal cytology				
Positive	68	28.7	34	30.9
Negative	169	71.3	76	69.1
Lymphadenectomy				
Yes	206	86.9	92	83.7
No	31	13.1	18	16.3
Lymph nodes metastasis				
Yes	60	25.3	14	12.7
No	146	61.6	96	87.2
Unknown	31	13.1	0	0.0
CA125(U/ml)				
<35	53	22.4	22	20.0
≥35	184	77.6	88	80.0
ALB [(g/L), mean (range)]	44.6(25.0-85.3)		42.9(27.2-54.8)	
Lymphocyte count [(10^9^/L), mean (range)]	1.5(0.1-3.2)		1.5(0.6-3.1)	
ALS				
Low (0)	83	35.1	32	29.0
High (1/2)	154	64.9	78	71.0

BMI, body-mass index; SMI, skeletal-muscle index; FIGO, International Federation of Gynecology and Obstetrics; ALB, albumin; ALS, albumin-lymphocyte score.

**Figure 1:**
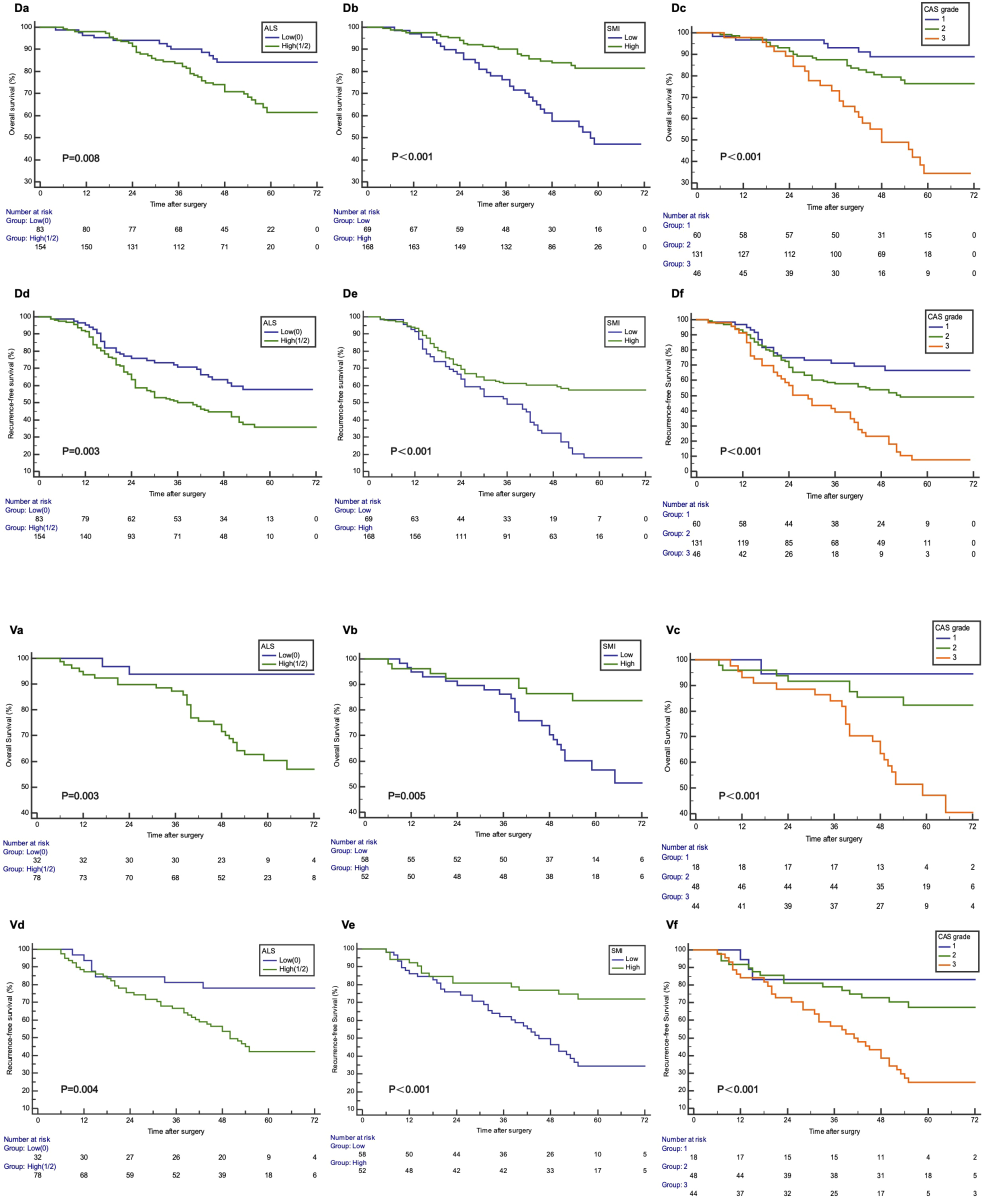
Kaplan–Meier curves for overall survival and recurrence-free survival stratified by ALS **(a, d)**, SMI **(b, e)**, and CAS **(c, f)** grade in the discovery (D) and validation cohort (V). ALS, albumin-lymphocyte score; SMI, skeletal muscle index; CAS, combination of albumin-lymphocyte score and skeletal muscle index.

**Figure 2:**
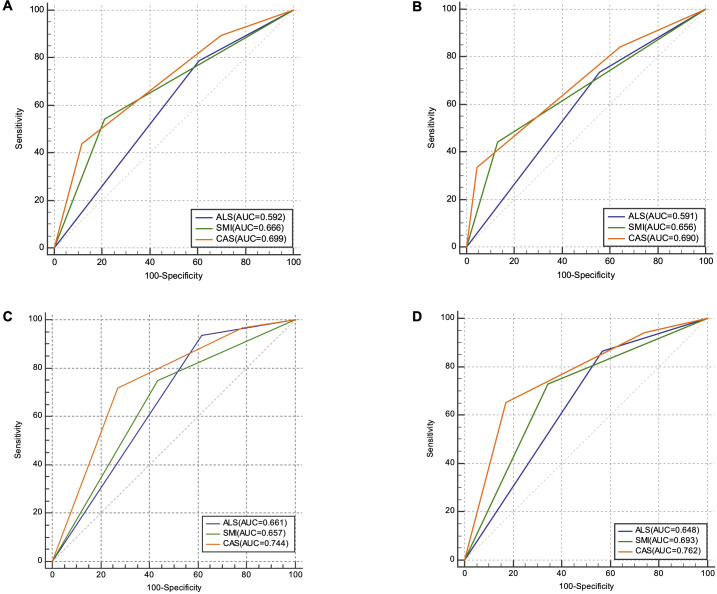
Comparison of area under receiver operator characteristic curves for ALS, SMI, and CAS grade in predicting overall survival and recurrence-free survival in the discovery **(A, B)** and validation cohort **(C, D)**. ALS, albumin-lymphocyte score; SMI, skeletal muscle index; CAS, combination of albumin-lymphocyte score and skeletal muscle index.

Kaplan–Meier survival curves indicated a significant correlation between elevated ALS and increased OS (P = 0.008, **Figure 1**. D-a) and RFS (P = 0.003, **Figure 1**. D-d). Furthermore, individuals with a high SMI exhibited notably improved OS (P < 0.001, **Figure 1**. D-b) and reduced postoperative recurrence (P < 0.001, **Figure 1**. D-e). According to the CAS grade, 60 patients (25.3%) were categorized as CAS grade 1, 131 (55.3%) as CAS grade 2, and 46 (19.4%) as CAS grade 3 (**Table 2**). Distinct CAS grades correlated significantly with BMI, FIGO stage, peritoneal cytology, and LMN. Kaplan–Meier curves indicated that patients with CAS grade 1 demonstrated the highest OS and RFS, whereas patients with CAS grade 3 demonstrated the poorest OS and RFS (P < 0.001, **Figure 1**. D-c/f). In terms of OS (**Figure 2A**), the AUC for the CAS grade (0.699) was significantly higher than that for ALS (0.592) and SMI (0.666). Similarly, for recurrence-free survival (RFS) (**Figure 2B**), the AUC for the CAS grade (0.690) was significantly higher than that for ALS (0.591) and SMI (0.656).

**Table 2:** Correlation between the CAS and clinicopathological characteristics.

Variable	CAS							
	Training cohort				Validation cohort			
	Grade 1	Grade 2	Grade 3	P-value	Grade 1	Grade 2	Grade 3	P-value
**Total patients**	60	131	46		18	48	44	
**Age** **[year, mean(range)]**	51.55 (23-75)	53.67 (18-78)	52.58 (33-75)	0.517	49.39 (35-63)	53.67 (18-78)	52.48 (33-75)	0.388
**BMI [mean(range)]**	24.39 (18.43-32.63)	23.02 (13.62-32.04)	20.80 (16.63-30.44)	**< 0.001**	24.41 (19.23-28.47)	23.25 (15.93-32.01)	22.36 (17.69-27.24)	**0.027**
Histology								
Serous	30	82	25	0.228	18	35	32	0.052
Others	30	49	21		0	13	12	
Grade								
1-2	15	29	7	0.463	5	14	12	0.923
3	45	102	39		13	34	32	
FIGO stage								
IA-IIB	36	47	20	**0.029**	14	23	14	0.932
III-IV	19	66	18		4	25	30	
Unknown	5	18	8		0	0	0	
Laterality								
Unilateral	45	81	33	**0.150**	15	33	30	0.446
Bilateral	15	50	13		3	15	14	
Residual disease								
<1cm	56	111	42	0.178	16	28	24	**0.026**
≥1cm	4	20	4		2	20	20	
Peritoneal cytology								
Positive	51	87	31	**0.025**	3	15	18	**0.006**
Negative	9	44	15		15	33	16	
Lymph nodes metastasis								
Yes	6	41	25	**< 0.001**	0	4	10	**0.007**
No	49	72	13		18	44	34	
Unknown	5	18	8		0	0	0	
CA125(U/ml)								
<35	19	24	10	0.120	5	12	5	0.597
≥35	41	107	36		13	36	39	
**ALB** **[(g/L), mean (range)]**	46.96 (43.90-80.30)	44.21 (31.60-85.30)	42.66 (25-51.10)	**< 0.001**	45.59 (41.10-50.10)	43.32 (31.70-54.80)	41.27 (27.20-50.00)	**< 0.001**
**Lymphocyte count [(10^9^/L), mean (range)]**	1.83 (1.34-3.15)	1.37 (0.14-2.96)	1.19 (0.69-2.27)	**< 0.001**	1.88 (1.39-3.10)	1.45 (0.70-2.40)	1.29 (0.60-2.50)	**< 0.001**
ALS								
Low (0)	60	23	0	**< 0.001**	18	14	0	**< 0.001**
High (1/2)	0	108	46		0	34	44	
**SMI [mean(range)]**	45.40 (38.53-64.51)	42.49 (27.83-67.52)	35.55 (30.58-38.47)	**< 0.001**	43.05 (38.65-53.67)	40.68 (27.99-52.24)	34.63 (24.58-45.77)	**< 0.001**

CAS, combination of albumin-lymphocyte score and skeletal muscle index; BMI, body-mass index; FIGO, International Federation of Gynecology and Obstetrics; ALB, albumin; ALS, albumin-lymphocyte score; SMI, skeletal-muscle index.

Bold values mean statistic significance.

In the training cohort, potential prognostic factors correlated with poor OS and RFS were identified using univariate Cox regression analyses. These factors included residual disease (OS HR: 2.956 (1.567–5.507), P = 0.001; RFS HR: 3.112 (1.975–4.904), P < 0.001), diminished ALS (OS HR: 2.320 (1.226–4.389), P = 0.01; RFS HR: 1.806 (1.206–2.707), P = 0.004), reduced SMI (OS HR: 0.325 (0.193–0.548), P < 0.001; RFS HR: 0.475 (0.332–0.680), P < 0.001), and elevated CAS grades (all P < 0.001). Other prognostic indicators, including residual disease, were found to have an influence not only on OS but also on RFS (**Table 3**). In contrast, BMI and postoperative chemotherapy did not significantly influence prognosis for OS or RFS. The multivariate Cox proportional hazards regression model (**Table 4**) was constructed based on FIGO stage, residual disease, LNM, and CAS grade. The independent risk factors for OS in patients with EOC were residual disease and elevated CAS grade. In addition, the independent variables for RFS were FIGO stage, residual disease, and elevated CAS grade.

**Table 3:** Univariate cox proportional hazards regressions identify prognostic factor for overall survival and recurrence-free survival.

Variables	Discovery cohort						Validation cohort					
	Overall survival			Recurrence-free survival			Overall survival			Recurrence-free survival		
	HR	95% CI	P	HR	95% CI	P	HR	95% CI	P	HR	95% CI	P
Age	1.027	1.002-1.052	**0.034**	1.012	0.996-1.028	0.145	1.015	0.984-1.047	0.35	1.018	0.994-1.044	0.148
BMI	0.999	0.922-1.083	0.983	0.984	0.931-1.039	0.565	0.908	0.799-1.032	0.141	0.918	0.833-1.014	0.092
Histology (Others vs. Serous)	1.127	0.669-1.899	0.653	0.542	0.371-0.792	**0.002**	0.59	0.227-1.533	0.279	0.618	0.301-1.269	0.19
FIGO stage (III-IV vs. I-II)	1.576	0.876-2.836	0.129	3.152	2.084-4.765	**<0.001**	4.134	1.786-9.569	**0.001**	3.262	1.787-5.956	**<0.001**
Laterality (Bilaterality vs. Unilaterality)	0.865	0.490-1.525	0.616	1.672	1.161-2.406	**0.006**	1.754	0.866-3.551	0.119	1.546	0.878-2.722	0.131
Residual disease	2.956	1.567-5.507	**0.001**	3.112	1.975-4.904	**<0.001**	4.11	1.738-9.716	**0.001**	3.357	1.732-6.508	**<0.001**
Peritoneal cytology (Positive vs. Negative)	1.22	0.698-2.133	0.485	1.881	1.300-2.722	**0.001**	1.589	0.784-3.218	0.199	1.407	0.799-2.477	0.237
LNM (Yes vs. No)	1.452	0.791-2.666	0.228	2.309	1.555-3.430	**<0.001**	2.709	1.212-6.055	**0.015**	2.372	1.214-4.635	**0.011**
CA125	0.739	0.410-1.334	0.316	1.636	1.022-2.619	**0.04**	1.0001	1.00001-1.0003	0.124	1.0001	0.999-1.000	0.185
ALS (Low vs. High)	2.32	1.226-4.389	**0.01**	1.806	1.206-2.707	**0.004**	6.593	1.575-27.589	**0.01**	3.007	1.355-6.670	**0.007**
SMI (Low vs. High)	0.325	0.193-0.548	**<0.001**	0.475	0.332-0.680	**<0.001**	0.334	0.150-0.745	**0.007**	0.343	0.186-0.636	**0.007**
CAS	2.735	1.815-4.121	**<0.001**	2.003	1.522-2.636	**<0.001**	3.474	1.783-6.770	**<0.001**	2.777	1.725-4.472	**<0.001**

HR, hazard ratio; BMI, body-mass index; FIGO, International Federation of Gynecology and Obstetrics; LNM, lymph nodes metastasis; ALS, albumin-lymphocyte score; SMI, skeletal-muscle index; CAS, combination of albumin-lymphocyte score and skeletal-muscle index.

Bold values mean statistic significance.

**Table 4:** Multivariate cox proportional hazards regressions identify prognostic factor for overall survival and recurrence-free survival.

Variables	Discovery cohort						Validation cohort					
	Overall survival			Recurrence-free survival			Overall survival			Recurrence-free survival		
	HR	95% CI	P	HR	95% CI	P	HR	95% CI	P	HR	95% CI	P
FIGO stage (III-IV vs. I-II)	1.412	0.686-2.908	0.349	3.299	2.006-5.429	**<0.001**	1.166	0.358-3.793	0.799	1.394	0.610-3.188	0.431
Residual disease	3.668	1.511-8.902	**0.004**	2.972	1.612-5.478	**<0.001**	4.006	1.403-11.441	**0.009**	2.997	1.388-6.469	**0.005**
LNM (Yes vs. No)	0.669	0.306-1.462	0.314	0.752	0.455-1.244	0.267	1.004	0.284-3.553	0.995	1.069	0.424-2.695	0.887
CAS	2.731	1.705-4.371	**<0.001**	2.415	1.729-3.373	**<0.001**	4.066	1.651-10.014	**0.002**	2.919	1.579-5.397	**<0.001**

HR, hazard ratio; FIGO, International Federation of Gynecology and Obstetrics; LNM, lymph nodes metastasis; CAS, combination of albumin-lymphocyte score and skeletal muscle index.

Bold values mean statistic significance.

### 3.2 Validation cohort

A total of 110 patients from our cooperative hospitals were enrolled in the validation cohort. The median patient age was 52.5 (18–78) years. The median BMI and SMI were 23.09 kg/m^2^ and 38.64 cm^2^/m^2^, respectively (**Table 1**). Consistent with the discovery cohort, distinct CAS grades correlated significantly with BMI, peritoneal cytology, and LNM. Patients with elevated ALS and SMI exhibited notably improved OS (ALS P = 0.003, **Figure 1**. V-a; SMI P < 0.001, **Figure 1**. V-b) and reduced postoperative recurrence (ALS P = 0.004, **Figure 1**. V-d; SMI P = 0.005, **Figure 1**.V-e). Likewise, patients with CAS grade 1 demonstrated the highest OS and RFS, whereas patients with CAS grade 3 demonstrated the poorest OS and RFS (both P < 0.001, **Figure 1**. V-c/f). For OS and RFS (**Figures 2C, D**), the AUC for CAS grade (OS: 0.744, RFS: 0.762) exhibited a statistically significant increase compared to ALS (OS: 0.661, RFS: 0.648) and SMI (OS: 0.657, RFS: 0.693). Moreover, univariate Cox analysis established that residual disease, diminished ALS, reduced SMI, and elevated CAS grades were correlated with adverse outcomes in terms of both OS and RFS (all P < 0.001; **Table 3**). Multivariate Cox regression analysis confirmed that residual disease and high CAS grade were independent variables for both OS and RFS (all P < 0.001; **Table 4**), which was consistent with the discovery cohort.

## 4 Discussion

This study proposes a novel composite index integrating albumin, lymphocyte count, and SMI to evaluate immunonutritional status in patients with ovarian cancer. Compared with conventional metrics such as BMI, the CAS grade provides a more comprehensive assessment of both nutritional and immune conditions. Our analysis demonstrated that the CAS grade, but not BMI, independently predicted unfavorable survival outcomes in patients undergoing debulking surgery. Patients with CAS grade 1 had the most favorable prognosis and the highest BMI, whereas those with CAS grade 3 exhibited the poorest OS and RFS.

Increasing evidence highlights the critical role of inflammation and immune responses in tumor development and progression (16). Nutritional status strongly influences inflammatory mediators and leukocyte function, thereby linking metabolism, immunity, and cancer progression (8). Lymphocytes play a key role in tumor cell clearance and antitumor immune responses. Metabolic regulation is also essential for immune function. For instance, enolase 1, a key glycolytic enzyme, supports T-cell antitumor activity, whereas tumor-infiltrating T cells often exhibit impaired glucose metabolism and reduced enolase 1 function (17, 18). Albumin, synthesized by hepatocytes, is widely recognized as a marker of nutritional status. Systemic inflammation suppresses albumin synthesis through proinflammatory cytokines such as interleukin-1, interleukin-6, and tumor necrosis factor-α, which simultaneously contribute to tumor progression (19). Sarcopenia, characterized by reduced skeletal muscle mass, has also been linked to insulin resistance (20) and immune dysfunction (21). Together, these mechanisms may promote tumor progression and adversely affect patient outcomes.

Inflammation-based scoring systems have therefore attracted increasing attention in OC research. Indicators such as the neutrophil-to-lymphocyte ratio (NLR) and platelet-to-lymphocyte ratio (PLR) have demonstrated prognostic value in several studies (22, 23). Cho et al. first reported preoperative NLR as an independent prognostic factor in OC (4), and subsequent studies have validated its predictive significance across different OC subtypes and treatment strategies. Meta-analyses have further shown that elevated PLR is associated with worse survival outcomes, with pooled hazard ratios of 1.66 for OS and 1.61 for RFS.

Growing recognition of the interplay between nutrition and inflammation has led to the development of immunonutritive scoring systems. Although markers such as the C-reactive protein–to–albumin ratio (24) and the prognostic nutritional index (PNI) (25) have been widely studied, a universally accepted immunonutritive standard remains lacking. Recently, a prognostic model integrating albumin and globulin was proposed for intrahepatic cholangiocarcinoma (26). Inspired by this approach, we developed a similar stratification system combining albumin and lymphocyte counts to generate a novel immunonutritive score for patients with OC.

Skeletal muscle is an important endocrine organ that secretes myokines and influences cancer outcomes (21). Multiple studies have reported an association between low skeletal muscle mass and poor prognosis in several malignancies (27, 28). However, its prognostic significance in OC remains controversial (29–31). Some studies found no significant relationship between sarcopenia and 5-year OS (30), whereas a recent meta-analysis reported that low SMI was associated with significantly worse survival (32). The CAS grade was therefore designed to enhance the prognostic value of SMI by integrating immunonutritional factors. In our study, the CAS grade independently predicted both RFS and OS, and its predictive performance exceeded that of ALS or SMI alone, suggesting improved prognostic stratification.

Several limitations should be considered. First, although our results were validated in independent cohorts, the cut-off values were derived using a data-driven approach, which may introduce overfitting and limit generalizability. Differences in body composition and nutritional status across populations may further influence the applicability of these thresholds. Prospective international multicenter studies involving ethnically diverse populations and different OC subtypes are therefore required to validate the robustness of the CAS grade. Second, CT imaging was not available for all patients because of cost or feasibility constraints, whereas serological markers were more readily obtainable. As a result, patients without CT scans were excluded from the analysis, which may have introduced selection bias. In addition, the present analysis focused exclusively on the baseline (preoperative) CAS grade and did not account for potential dynamic changes during treatment and disease progression. Third, the relatively short follow-up duration may limit assessment of the long-term predictive performance of the CAS grade. Longer follow-up will be necessary to confirm the durability of its prognostic value. Moreover, certain potentially relevant confounders were not captured in this study, including comorbidities affecting nutritional or muscle status and detailed information on chemotherapy intensity or treatment completion. These unmeasured factors may contribute to residual confounding. Finally, because the primary aim of this study was to develop and validate the CAS grade, clinical data on other established prognostic indicators—such as performance status scales (e.g., ECOG or Karnofsky scores)—were not systematically collected. Similarly, detailed immune profiling, including immune cell subset analysis, inflammatory cytokine measurement, and tumor immune microenvironment characterization, was not available. Consequently, direct comparisons between CAS grade and these established markers were not feasible.

In conclusion, this study provides a comprehensive evaluation of prognostic indices in OC by integrating both nutritional and immune parameters. The CAS grade demonstrated superior prognostic performance compared with conventional indicators. The index was first developed in the West China Second Hospital cohort and subsequently validated in an independent multi-institutional cohort. Importantly, SMI and ALS were determined using the most recent imaging examinations and preoperative laboratory data while excluding intraoperative and postoperative factors to minimize potential surgical influences. Future studies should investigate longitudinal changes in CAS grade through serial monitoring, which may further improve prognostic assessment and clinical decision-making in OC.

## Data Availability

The raw data supporting the conclusions of this article will be made available by the authors, without undue reservation.
